# Droplet Digital PCR Analysis of Liquid Biopsy Samples Unveils the Diagnostic Role of hsa-miR-133a-3p and hsa-miR-375-3p in Oral Cancer

**DOI:** 10.3390/biology9110379

**Published:** 2020-11-06

**Authors:** Salvatore Crimi, Luca Falzone, Giuseppe Gattuso, Caterina Maria Grillo, Saverio Candido, Alberto Bianchi, Massimo Libra

**Affiliations:** 1 Department of General Surgery, Section of Maxillo Facial Surgery, Policlinico San Marco, University of Catania, 95123 Catania, Italy; torecrimi@gmail.com (S.C.); alberto.bianchi@unict.it (A.B.); 2 Epidemiology Unit, IRCCS Istituto Nazionale Tumori “Fondazione G. Pascale”, 80131 Naples, Italy; 3 Department of Biomedical and Biotechnological Sciences, University of Catania, 95123 Catania, Italy; peppeg9305@gmail.com (G.G.); scandido@unict.it (S.C.); m.libra@unict.it (M.L.); 4 Otolaryngology Unit, Department of Medical Sciences, Surgical and Advanced Technologies, University of Catania, 95123 Catania, Italy; grillo.caterinamaria@gmail.com; 5 Research Center for Prevention, Diagnosis and Treatment of Cancer, University of Catania, 95123 Catania, Italy

**Keywords:** oral cancers, microRNAs, ddPCR, diagnosis, biomarkers, cancer, epigenetics

## Abstract

**Simple Summary:**

Despite the availability of screening programs, oral cancer is often diagnosed due to the lack of effective biomarkers. Therefore, the identification of new effective diagnostic and late prognostic biomarkers is of fundamental importance for the management of this tumor type. In our previous computational study, we have identified a set of microRNAs (miRNAs) significantly dysregulated in oral cancer and with a potential diagnostic and prognostic significance for oral cancer patients. Starting from our preliminary bioinformatics results, the aim of the present study was to validate the diagnostic potential of four selected miRNAs, hsa-miR-133a-3p, hsa-miR-375-3p, hsa-miR-503-5p and hsa-miR-196a-5p, in liquid biopsy samples obtained from oral cancer patients and healthy donors. For this purpose, the expression levels of the selected miRNAs were determined in plasma samples by using specific miRNA probes and droplet digital PCR (ddPCR). The ddPCR results showed that the hsa-miR-133a-3p and hsa-miR-375-3p were significantly down-regulated in oral cancer and their evaluation in liquid biopsy samples can predict the risk of oral cancer development with high sensitivity and specificity. Finally, the computational analysis of miRNA expression and clinical-pathological features of patients allowed us to establish the functional role and prognostic significance of the two validated miRNAs.

**Abstract:**

Despite the availability of screening programs, oral cancer deaths are increasing due to the lack of diagnostic biomarkers leading to late diagnosis and a poor prognosis. Therefore, there is an urgent need to discover novel effective biomarkers for this tumor. On these bases, the aim of this study was to validate the diagnostic potential of microRNAs (miRNAs) through the analysis of liquid biopsy samples obtained from ten oral cancer patients and ten healthy controls. The expression of four selected miRNAs was evaluated by using droplet digital PCR (ddPCR) in a pilot cohort of ten oral cancer patients and ten healthy donors. Bioinformatics analyses were performed to assess the functional role of these miRNAs. The expression levels of the predicted down-regulated hsa-miR-133a-3p and hsa-miR-375-3p were significantly reduced in oral cancer patients compared to normal individuals while no significant results were obtained for the up-regulated hsa-miR-503-5p and hsa-miR-196a-5p. ROC analysis confirmed the high sensitivity and specificity of hsa-miR-375-3p and hsa-miR-133a-3p. Therefore, both miRNAs are significantly down-regulated in cancer patients and can be used as biomarkers for the early diagnosis of oral cancer. The analysis of circulating miRNAs in a larger series of patients is mandatory to confirm the results obtained in this pilot study.

## 1. Introduction

Oral cancer represents one of the most prevalent tumors of the digestive tract, accounting for more than 350,000 novel diagnoses and 177,000 deaths every year [1]. Due to the lack of diagnostic biomarkers, a significant fraction of oral cancer lesions is diagnosed in an advanced stage when the tumor is not responsive to treatments [2]. Therefore, despite the availability of screening programs and novel anticancer therapies, the mortality rate of this tumor remains high [3,4].

As described for other tumors, several studies have demonstrated that the development of oral cancer is strictly related to numerous modifiable factors, including environmental and lifestyle risk factors, underlying the neoplastic transformation of cells [5,6]. Among these risk factors, the most frequently associated with oral cancer are alcohol intake and smoking [7,8]. Other causative agents of oral cancer are some microbial infections mainly due to Epstein Barr virus (EBV), human papillomaviruses (HPVs) or *Candida albicans* infection responsible for the activation of prooncogenic stimuli [9,10,11]. In this context, recent reviews of the literature have highlighted a double role of microbial infections and microbiota composition in preventing or favoring the onset of tumors, including that of the oral cavity [12,13]. All these risk factors are responsible for the development of both genetic and epigenetic alterations that can promote tumor development and progression by altering key cellular mechanisms, such as apoptosis or cell proliferation [14,15]. In the case of oral cancer, such risk factors were associated with the mutations and changes of expression genes involved in the EGFR epidermal growth factor pathway (including *TGF-β*, fibroblastic growth factor-BP (*FGF-BP*) and *MMK6*) and genes, including *CDKN2A*, *TP53*, *SYNE1*, *NOTCH1*, and *PIK3CA* notoriously involved in different tumors [16,17].

More recently, it was also demonstrated that environmental and lifestyle factors, including diet, exercise, alcohol consumption and smoking, are able to alter the methylation status of DNA and the expression levels of microRNAs (miRNAs) functionally related to oncogenes and tumor suppressor genes [18,19,20,21]. In this context, miRNAs, a class of small non-coding RNA of about 22 nucleotides are representing novel candidate biomarkers for different pathologies thanks to their abilities to interact with the 3′UTR region of targeted mRNAs by blocking or repressing their expression [22,23].

In order to find novel effective biomarkers for the management of oral cancer patients, our research group has previously identified a set of miRNAs potentially involved in the development and progression of oral cancer through the integrated analysis bioinformatics data contained in different miRNA expression datasets [24]. Among these miRNAs, the most altered in oral cancer patients and predictive for tumor aggressiveness were the two up-regulated miRNAs hsa-miR-503-5p and hsa-miR-196a-5p and the two down-regulated miRNAs hsa-miR-133a-3p and hsa-miR-375-3p.

Starting from our previous bioinformatics analyses, in the present study, we wanted to propose a novel strategy for oral cancer diagnosis based on the analysis of miRNAs expression levels in liquid biopsy samples. In particular, the diagnostic potential of hsa-miR-503-5p, hsa-miR-196a-5p, hsa-miR-133a-3p and hsa-miR-375-3p was assessed on liquid biopsy samples obtained from a pilot series of ten oral cancer patients and ten healthy individuals. For this purpose, we used the high-sensitive droplet digital PCR (ddPCR) system as, at present, it represents the best methods for the analysis of circulating or low-expressed miRNAs [25,26]. Although performed on a limited number of patients and normal individuals, the validation of the diagnostic potential of these miRNAs will pave the way to the development of novel effective strategies for the early diagnosis of oral cancer by using ddPCR and liquid biopsy samples obtained from patients with suspicious lesions or with an increased risk of oral cancer development.

## 2. Materials and Methods

### 2.1. Samples Included in the Study

A blinded pilot case series of ten oral cancer patients and ten healthy donors was included in the study in order to validate the prognostic value of hsa-miR-133a-3p, hsa-miR-375-3p, hsa-miR-196a-5p and hsa-miR-503-5p previously identified as key miRNAs involved in oral cancer development. For each individual enrolled in the study, two peripheral blood samples were collected, of which one containing anticoagulant (K3EDTA) (Becton, Dickinson and Company, Franklin Lakes, NJ, USA) for the collection of plasma, buffy coat and red cells, and the other one containing a separator gel for the collection of serum. Briefly, both blood samples were centrifuged at 2000 g for 10 min at room temperature in order to separate blood components. The blood vial containing the separator gel was used for the separation of serum thus obtaining about 3 mL of serum divided into three aliquots of 1 mL for each individual enrolled in the study. Similarly, the blood vial containing K3EDTA was used for the separation of plasma (about 2 mL), buffy coat (white blood cells) and red cells. The socio-demographic and clinical characteristics of the patients analyzed are reported in Table 1 (Table 1).

### 2.2. RNA Extraction and miRNAs Retrotranscription

Circulating total RNAs, including miRNAs, were extracted from plasma samples. Briefly, plasma samples were centrifuged at 2000 g × 10 min at room temperature in order to pool down debris and protein aggregates. Then 200 µL of plasma were extracted by using the miRNeasy Serum/Plasma Kit (Cat. N. 217184, Qiagen, Hilden, Germany) by adding the spike-in control cel-miR-39 as exogenous control (Cat. N. 219610, Qiagen, Hilden, Germany).

After RNA extraction, miRNAs were retrotranscribed into cDNA by using TaqMan™ Advanced miRNA cDNA Synthesis Kit (Cat. N. A28007, Thermo Fisher Scientific, Waltham, MA, USA) following the manufacturer’s instructions. Briefly, first the 3’ poly-A tailing of miRNAs was performed and then the adaptor sequence was tied at the 5′ terminus of miRNAs. Subsequently, all mature miRNAs were reverse transcribed using universal primers able to bind the 5′ adaptor and the 3’ poly-A tail. Finally, the cDNA was amplified using the miR-Amp Master Mix and the Universal miR-Amp primers (Thermo Fisher Scientific, Waltham, MA, USA).

### 2.3. Analysis of miRNAs Expression Through Droplet Digital PCR

The cDNA previously obtained was analyzed by using specific TaqMan Advanced probes for the detection of the four selected miRNAs hsa-miR-503-5p, hsa-miR-196a-5p, hsa-miR-133a-3p, hsa-miR-375-3p, and for the spike-in control cel-miR-39-3p (TaqMan™ Advanced miRNA Assay, Cat. N. A25576, Thermo Fisher Scientific, Waltham, MA, USA) using a custom ddPCR protocol. Briefly, the ddPCR reaction mix was prepared by using 11 µL of 2x ddPCR Supermix for Probes (No dUTP) (Cat. N. 1863024, Bio-Rad, Hercules, CA, USA), 1.1 µL of miRNA specific TaqMan probe (A25576, Thermo Fisher Scientific, Waltham, MA, USA), 4.9 µL of RNase/DNase-free water and 5 µL of cDNA in order to obtain a final volume of 22 µL. Subsequently, twenty microliters of the reaction mix were used to generate droplets with the QX200 droplet generator (Bio-Rad). After generation, the droplets were transferred into a 96-well plate, sealed and amplified in a C1000 Thermal Cycler (Bio-Rad) under the following thermal conditions: Polymerase activation at 95 °C for 10 min, 40 cycles of amplification at 94 °C for 30 s (denaturation) and 60 °C for 1 min (annealing), droplets stabilization at 98 °C for 10 min followed by an infinite hold at 4 °C. A ramp rate of 2 °C/s was used among the steps of the amplification. After amplification, positive and negative droplets were read in the QX200 Droplet Reader (Bio-Rad). All experiments were performed in triplicate. The expression levels of miRNAs were normalized according to the expression levels of the exogenous spike-in cel-miR-39-3p in order to avoid differences in miRNA expression due to non-efficient extraction.

### 2.4. Bioinformatics Analyses

In order to better clarify the involvement of hsa-miR-133a-3p and hsa-miR-375-3p in oral cancer development and aggressiveness, different computational approaches have been used. Firstly, the targeted genes of the two validated miRNAs were identified using the bioinformatics tool miRWalk v2.0 (http://mirwalk.umm.uni-heidelberg.de) [27]. In particular, miRWalk is able to identify the genes targeted by both hsa-miR-133a-3p and hsa-miR-375-3p by analyzing interaction data contained in 14 different resources for miRNA-target data, including miRBase, TargetScan, KEGG pathway, etc.

For the targeted genes identified by miRWalk, the protein-protein interaction (PPI) and the biological and molecular functions were assessed by using, respectively, the Search Tool for the Retrieval of Interacting Genes/Proteins (STRING v11.0) and GO PANTHER v15.0 software [28,29]. In addition, GEPIA and miRCancerdb tools were used to establish if the genes targeted by hsa-miR-133a-3p and hsa-miR-375-3p are differentially expressed in oral cancer patients compared to healthy controls and if the two down-regulated miRNAs are negatively or positively correlated with the expression levels of the genes identified through miRWalk. Both tools analyze the data contained in The Cancer Genome Atlas Head and Neck Cancer (TCGA HNSC) database [30,31].

Finally, the clinical implication of the two down-regulated miRNAs was assessed by analyzing the clinical data contained in TCGA HNSC database and downloaded by using the online exploration tool UCSC Xena Browser [32].

### 2.5. Statistical Analyses

For the absolute quantification of miRNAs expression in plasma samples of oral cancer patients and normal individuals, the QuantaSoft software was used (Bio-Rad). Kolmogorov-Smirnov normality test was performed to assess the distribution of hsa-miR-133a-3p and hsa-miR-375-3p expression levels observed with ddPCR and for the expression levels of these miRNAs deposited on the TCGA HNSC database. Statistical differences between cases and controls were evaluated by using unpaired Student t-test. Receiver operating characteristic (ROC) curves were obtained by using GraphPad Prism v.6 in order to evaluate the specificity and sensitivity of the analyzed miRNAs.

Kruskal-Wallis test (and post-hoc Dunn’s Multiple Comparison test) and One-way ANOVA test (and post-hoc Dunnett’s Multiple Comparison test) were used for assessing the statistical differences existing between the expression levels of hsa-miR-375-3p and hsa-miR-133a-3p reported in the TCGA HNSC database according to the oral cancer tumor stages. All statistical analyses were performed by using GraphPad Prism v.6.

## 3. Results

### 3.1. Analysis of miRNA Expression Levels in Liquid Biopsy Samples of Oral Cancer Patients and Healthy Controls

In the present study, a pilot case series of 20 individuals, of which ten were oral cancer patients and ten were healthy controls, was analyzed. The ten oral cancer patients enrolled in the study had a diagnosis of oral squamous cell carcinoma (OSCC). The clinical validation of the diagnostic potential of oral cancer-related miRNAs previously identified [24] was performed on liquid biopsy samples collected and stored as described in Materials and Methods section. In particular, two up-regulated miRNAs, hsa-miR-503-5p and hsa-miR-196a-5p, and two down-regulated miRNAs, hsa-miR-375-3p and hsa-miR-133a-3p, were analyzed by using the innovative high-sensitive ddPCR amplification system. Although performed in a low number of samples (ten tumor samples and ten normal controls), statistically significant results were obtained by analyzing the absolute quantitative of these miRNAs.

In particular, positive signals were obtained for three out of the four selected miRNAs thus allowing their absolute quantification in both tumor and normal samples. The TaqMan Advanced probe specific for hsa-miR-196a-5p does not give good quality signals, therefore this miRNA was excluded from the study.

**Figure 1:**
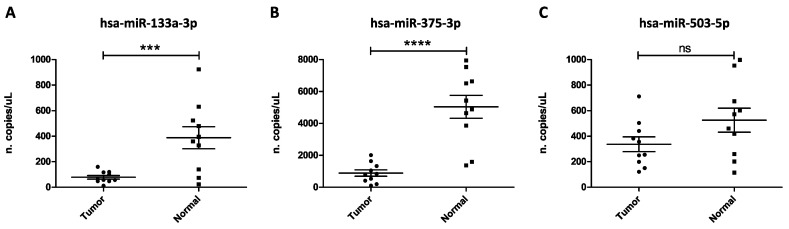
(**A**) Plasma levels of hsa-miR-133a-3p in oral cancer patients vs normal controls; (**B**) Plasma levels of hsa-miR-375-3p in oral cancer patients vs normal controls; (**C**) Plasma levels of hsa-miR-503-5p in oral cancer patients vs normal controls. Results are expressed as copies/µL which means the number of copies of a target contained in 1 μL of the 22 μL reaction mix. ***: *p* < 0.001; ****: *p* < 0.0001; ns: no statistical significance.

The most important results were obtained for the down-regulated miRNAs hsa-miR-133a-3p and hsa-miR-375-3p (Figure 1A,B), while, despite the good amplification signals obtained, no statistical difference was observed for the expression levels of the up-regulated hsa-miR-503-5p between tumor and normal samples (Figure 1C). As shown in Figure 1, the circulating levels of the predicted down-regulated miRNAs hsa-miR-133a-3p and hsa-miR-375-3p were effectively lower in tumor samples compared to normal individuals with *p*-values of *p* < 0.001 and *p* < 0.0001, respectively (Figure 1A,B).

### 3.2. Diagnostic Value of hsa-miR-133a-3p and hsa-miR-375-3p

**Figure 2:**
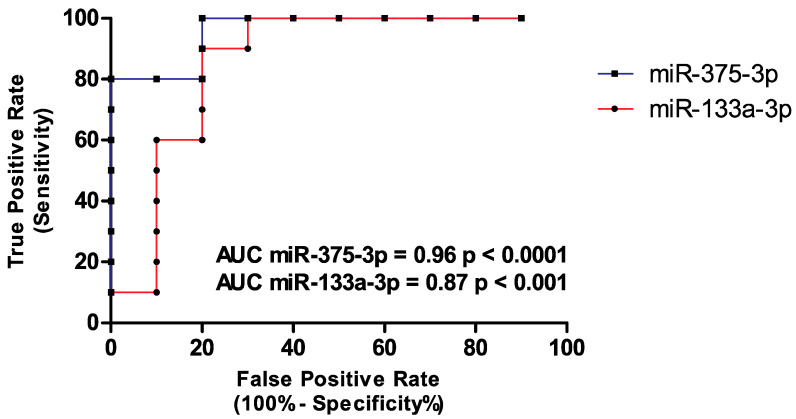
ROC analysis demonstrated the high diagnostic value of both miRNAs (hsa-miR-375-3p 80% sensitivity and 100% specificity; hsa-miR-133a-3p 90% sensitivity and 80% specificity).

The absolute quantification of hsa-miR-133a-3p and hsa-miR-375-3p has demonstrated the strong down-regulation of these miRNAs in oral cancer patients, thus confirming the results obtained by our research group in a previous bioinformatics study [24]. To further assess the specificity and sensitivity of these miRNAs and their role as novel promising diagnostic biomarkers for oral cancer, ROC curves were calculated. ROC analysis revealed high specificity and sensitivity rates for both miRNAs. In particular, the sensitivity and specificity for hsa-miR-133a-3p were 90% and 80%, respectively (AUC = 0.8600; 95% CI 0.6757 to 1.044) while for hsa-miR-375-3p were 80% and 100% respectively (AUC = 0.9600; 95% CI 0.8832 to 1.037). All the data were statistically significant (*p* < 0.01 for hsa-miR-133a-3p and *p* < 0.001 for hsa-miR-375-3p) (Figure 2).

### 3.3. Functional Role of hsa-miR-133a-3p and hsa-miR-375-3p in Oral Cancer

The bioinformatics analyses performed for the two down-regulated miRNAs hsa-miR-133a-3p and hsa-miR-375-3p revealed the strict clinical implication of these miRNAs in the pathogenesis and aggressiveness of oral cancer.

**Figure 3:**
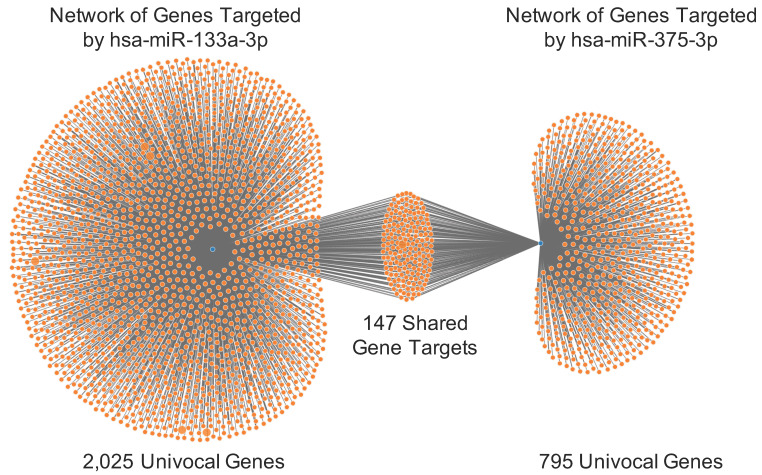
Network of genes targeted individually or in common by hsa-miR-133a-3p and hsa-miR-375-3p.

First, miRWalk analysis allowed the identification of the genes targeted by hsa-miR-133a-3p and hsa-miR-375-3p. In particular, the analysis revealed that a total of 2,025 univocal genes are targeted by hsa-miR-133a-3p, while 795 univocal genes are targeted by hsa-miR-375-3p. The analyses revealed also that some of these genes are targeted in different gene positions, suggesting that for some genes the regulatory action of miRNAs is stronger (data not shown). By merging the lists of targeted genes obtained for the two down-regulated miRNAs, a panel of 147 univocal genes targeted by both hsa-miR-133a-3p and hsa-miR-375-3p was identified (Figure 3).

As already mentioned, some of these 147 genes were targeted in different gene positions. In particular, the genes with more miRNAs binding site were *LRRC20* (13 sites), *NPNT* (12 sites), *TP53* (11 sites), *PRR29* (ten sites), *DLG2* (nine sites), *TLE3*, *ACSL6*, *CD84*, *KAT5*, *PTPRN2*, *QTRT2*, *RAP1GAP* and *SERF2* (eight sites) (data not shown).

**Figure 4:**
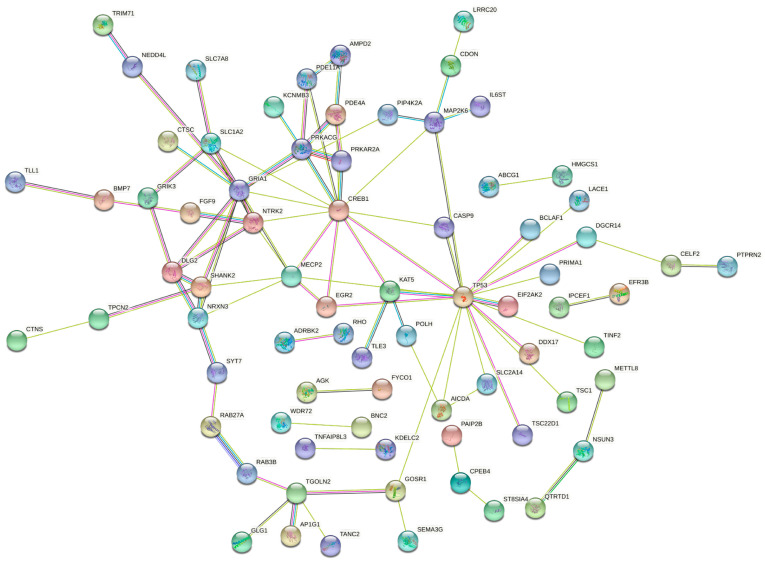
STRING protein interaction network of 75 out of 147 genes targeted by both hsa-miR-133a-3p and hsa-miR-375-3p.

By using STRING, the PPI interactions among the 147 genes targeted by both miRNAs were evaluated. As reported in Figure 4, STRING analysis revealed that of the 147 genes only 75 were interconnected with each other. Of these genes, the most interconnected were *TP53*, *CREB1*, *NTK2*, *DLG2* and *GRIA1* (Figure 4).

As regards the functional and molecular functions of the 147 miRNA-targeted genes, the GO PANTHER analysis showed that the majority of these genes are involved in binding (29 genes) and catalytic (29 genes) activities (Figure S1); within the category “Biological process”, the majority of genes were involved in the regulation of cellular processes (51 genes) and metabolic processes (31 genes) as well as in biological regulation (34 genes) (Figure S2). In addition, GO PANTHER analysis gave information about the protein classes of the 147 genes showing that the majority of genes encodes for gene-specific transcriptional regulators (11 genes), nucleic acid binding proteins (ten genes) and protein modifying enzymes (eight genes) (Figure S3).

Subsequently, to establish if the hsa-miR-133a-3p and hsa-miR-375-3p target genes are differentially expressed in oral cancer patients compared to healthy controls, GEPIA and miRCancerdb tools were used. As shown in Table 2, of the 147 gene targets only 14 were significantly dysregulated in oral cancer patients compared to healthy individuals. Of these 14 genes, ten are over-expressed, suggesting that the up-regulation of these genes in oral cancer may be related to the down-regulation of hsa-miR-133a-3p and hsa-miR-375-3p and, consequently, to the lack of the inhibitory action of these two miRNAs (Table 2, Figure S4). These data were further confirmed by miRCancerdb analysis that showed weak-moderate positive correlations between the two down-regulated miRNAs and three of the four down-regulated genes (*DLG2*, *CPEB4*, *PAIP2B*), and weak negative correlations between the two down-regulated miRNAs and three of the ten significantly up-regulated genes in oral cancer (*HOXC11*, *EIF2AK2*, *CTSC*) (Table 2).

**Figure 5:**
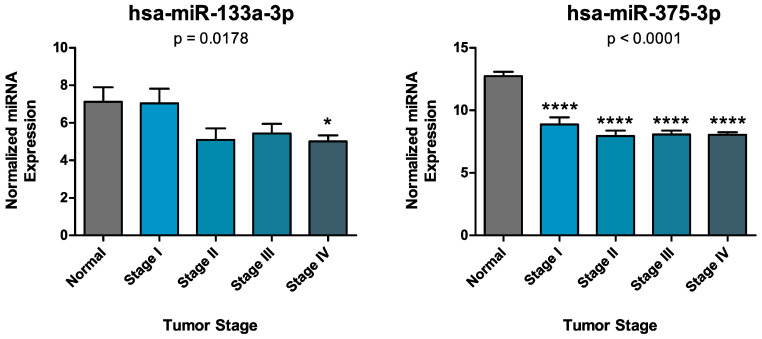
Panel (**A**) Statistical difference of hsa-miR-133a-3p expression in normal individuals and different tumor stages according to the TCGA HNSC data (*p* = 0.0178); Panel (**B**) Statistical difference of hsa-miR-375-3p expression in normal individuals and different tumor stages according to the TCGA HNSC data (*p* < 0.0001). *: *p* < 0.05; ****: *p* < 0.0001.

Finally, the analysis of miRNAs expression according to the clinical-pathological data contained in the TCGA HNSC database revealed that the expression levels of hsa-miR-133a-3p and hsa-miR-375-3p decrease significantly in the group of patients with more advanced tumors. Overall, the expression levels of hsa-miR-133a-3p differ significantly among all groups analyzed (Kruskal-Wallis test *p* = 0.0178). In addition, the post-hoc Dunn’s Multiple Comparison test highlighted a statistical difference between normal individuals and Stage IV oral cancer patients for this miRNA. As regards the expression levels of hsa-miR-375-3p, One-way ANOVA test showed a statistical reduction of this miRNA among groups (*p* < 0.0001) while the post-hoc Dunnett’s Multiple Comparison test revealed that the expression levels of hsa-miR-375-3p were significantly lower in all tumor stages compared to the expression levels observed in normal individuals (Figure 5). 

These data suggest that the evaluation of hsa-miR-375p may be useful to early diagnose low-grade oral cancer, while the concomitant evaluation of both hsa-miR-133a-3p and hsa-miR-375-3p may be predictive of worse prognosis when the expression levels of these miRNAs are significantly low. Regarding the expression levels of the two validated miRNAs according to the lymph node involvement, the paired and unpaired t-tests performed for hsa-miR-133a-3p and hsa-miR-375-3p, respectively, do not reveal any statistical differences between oral cancer patients with positive lymph nodes (N+) compared to patients without lymph node involvement (N0) (Figure S5).

## 4. Discussion

Several studies have tried to identify novel diagnostic and prognostic biomarkers for the management of oral cancer patients, however, the currently available diagnostic strategies, mainly based on the evaluation of tumor biomarkers (CEA, CA19-9, CA125) or serum and salivary pro-inflammatory cytokines (IL-6, IL-8, etc.), often fail to diagnose correctly oral cancer lesions due to the low rates of sensitivity and specificity of these biomarkers [33,34,35]. 

In order to find novel effective biomarkers, several studies have tried to study the predictive value of several epigenetic factors for oral cancer, including DNA methylation hotspots and the alteration of non-coding RNAs (ncRNAs), however, these identified biomarkers are still under validation [36,37,38,39,40,41]. In this scenario, to the best of our knowledge, our research group was the first to comprehensively analyze all the bioinformatics data about miRNAs expression in oral cancer patients through the integrated analysis of miRNAs expression datasets obtained from Gene Expression Omnibus DataSets and The Cancer Genome Atlas databases [24]. In this study, we have identified a set of miRNAs that, according to rigorous bioinformatics and computational prediction analyses, was strictly involved in the development and aggressiveness of oral cancer. Among these miRNAs, the two down-regulated miRNAs hsa-miR-133a-3p and hsa-miR-375-3p and the two up-regulated miRNAs hsa-miR-503-5p and hsa-miR-196a-5p were the most altered in oral cancers and with the highest predictive value for oral cancer diagnosis [24]. 

To further confirm the diagnostic value of these four miRNAs previously identified in silico, validation analyses were here performed by assessing the expression levels of these miRNAs in liquid biopsy samples obtained from oral cancer patients and healthy donors. For this purpose, the high-sensitive ddPCR was used to analyze the expression levels of hsa-miR-133a-3p, hsa-miR-375-3p, hsa-miR-503-5p and hsa-miR-196a-5p selected as candidate diagnostic biomarkers for oral cancer. As miRNAs may be weakly expressed in liquid biopsy samples, ddPCR was used because it represents the best technique for the analysis of liquid biopsy samples and targets with low concentrations, including low amount of viral and bacterial nucleic acids as well as circulating tumor DNA and circulating miRNAs associated with other tumors [25,42,43,44].

The ddPCR analysis here performed by using TaqMan Advanced probes specific for the selected miRNAs has revealed that the expression levels of the predicted down-regulated miRNAs hsa-miR-133a-3p and hsa-miR-375-3p were significantly lower (*p* < 0.001 and *p* < 0.0001, respectively) in oral cancer patients compared to healthy controls. As regards the two predicted up-regulated miRNAs, the miR-196a-5p does not give results of good quality, therefore was excluded from the analysis, while the expression levels of miR-503-5p were not statistically different between normal and tumor samples, probably due to the low number of samples analyzed.

Noteworthy, although the low number of samples analyzed, the diagnostic value of the down-regulated miRNAs hsa-miR-133a-3p and hsa-miR-375-3p was strongly validated. Indeed, the ROC analysis showed that the evaluation of these two miRNAs, alone or in combination, ensures sensitivity and specificity rates ranging from 80% to 100%. Thus, the ddPCR evaluation of these miRNAs in liquid biopsy samples may predict the presence of oral cancer with great accuracy, avoiding false-negative or false-positive results.

The experimental data here obtained were further confirmed by performing computational analyses of publicly available miRNA-gene interaction and miRNAs expression databases, including miRWalk, TCGA HNSC database and miRCancerdb. These bioinformatics analyses demonstrated the inhibitory action of hsa-miR-133a-3p and hsa-miR-375-3p towards a total of 147 genes of which some are involved in several molecular and biological processes underlying tumor development, including TP53, SHANK2, CASP9, etc., whose dysregulation is notoriously observed in oral cancer [45,46,47].

Taking into account the gene expression and miRNA expression data contained in the TCGA HNSC database we further demonstrated that 14 of the 147 genes targeted by the two miRNAs were significantly dysregulated in oral cancer patients. Of these, ten were up-regulated in oral cancer probably due to the down-regulation of hsa-miR-133a-3p and hsa-miR-375-3p. In addition, the expression of some of these genes was also positively or negatively correlated (weak-moderate correlations) with the expression levels of miRNAs, suggesting the effective regulatory action of these miRNAs towards dysregulated genes in oral cancer.

Finally, the analysis of clinical-pathological and miRNAs expression data of oral cancer patients obtained from the database TCGA HNSC further confirmed the strong down-regulation of both hsa-miR-133a-3p and hsa-miR-375-3p in oral cancer patients, especially in those patients with advanced tumors.

Overall, the results of the present study confirmed the bioinformatics results previously obtained [24]. Indeed, despite the low number of samples analyzed, both hsa-miR-133a-3p and hsa-miR-375-3p were significantly down-regulated in oral cancer patients. In addition, the use of ddPCR for the analysis of liquid biopsy samples allowed the identification of slight differences of miRNAs expression among samples ensuring high sensitivity and specificity rates. Therefore, this pilot study represents a milestone for the implementation of novel diagnostic strategies based on the use of ddPCR for the analysis of liquid biopsy samples and the identification of diagnostic and prognostic biomarkers for oral cancer.

At present, the evaluation of miRNAs, including hsa-miR-133a-3p and hsa-miR-375-3p, has been performed mainly on oral cancer specimens just to confirm the presence of tumor-associated miRNAs and not to early diagnose neoplastic lesions [48,49,50,51]. For the first time, ddPCR is here applied for the analysis of liquid biopsy samples and the development of novel non-invasive diagnostic strategies based on the evaluation of miRNAs associated with precancerous lesions or early stage oral cancers. Indeed, it was widely demonstrated that epigenetic modifications represent early events of neoplastic transformation. In this context, the analysis of DNA methylation and miRNAs expression has been proposed for the early diagnosis of different tumors [52,53,54,55]. In line with the results achieved in this study, other studies demonstrated the diagnostic potential of both hsa-miR-133a-3p and hsa-miR-375-3p in different tumors, including gastric cancer, prostate cancer, hepatocellular carcinoma, colorectal cancer, etc., demonstrating the involvement of these miRNAs in cancer development, including that of oral cancer [56,57,58,59,60].

In conclusion, the innovative results obtained in the present study represent the starting point for the development of novel effective strategies for the management of oral cancer patients. Further experimental and functional studies on a large number of samples are needed in order to evaluate the expression levels of the miRNAs here identified and to further validate their predictive role as biomarkers for oral cancer.

## 5. Conclusions

Overall, in the present study, we identified two miRNAs, hsa-miR-133a-3p and hsa-miR-375-3p, whose down-regulation is predictive for oral cancer development and for a worse prognosis. Besides the diagnostic and prognostic potential of these miRNAs here validated, bioinformatics analyses revealed the molecular pathways and targeted genes of the two miRNAs thus highlighting also their effective involvement in oral cancer pathogenesis and aggressiveness. Surely, this study represents the starting point on which to develop confirmatory studies carried out on a wider cohort of patients. Indeed, despite the small number of patients here analyzed, the statistically significant results obtained pave the way to the development of novel diagnostic strategies based on the use of ddPCR and liquid biopsy for the analysis of miRNAs used as diagnostic and prognostic biomarkers for the management of oral cancers.

## Figures and Tables

**Table 1:** Clinical pathological features of patients.

Patients’ Characteristics	Cancer Patients (N. 10)		Normal Controls (N. 10)	
	N.	%	N.	%
**Sex**				
Male	6	60	9	90
Female	4	40	1	10
**Age**				
<45	0	0	0	0
45–59	3	30	6	60
>60	7	70	4	40
**Smoke**				
Yes	4	40	3	30
No	4	40	5	50
Ex-smoker	2	20	2	20
**Alchol**				
Yes	3	30	1	10
No	7	70	9	90
**Family History**				
Yes	6	70	3	30
No	4	30	7	70
**Tumor Stage**				
T1	1	10	NA	
T2	2	20		
T3	2	20		
T4	5	50		
**Lymph Nodes**				
Positive	2	20	NA	
Negative	8	80		
**Recurrence**				
Yes	3	30	NA	
No	7	70		

**Table 2:** Dysregulation and correlation levels of genes targeted by hsa-miR-133a-3p and hsa-miR-375-3p according to TCGA HNSC data.

Gene	GEPIA Analysis		miRCancerdb Correlation	
	Log2FC	Adj. *p*-Value	hsa-miR-133a-3p	hsa-miR-375-3p
*DLG2*	−1.259	5.55 × 10^−51^	0.19	0.34
*CPEB4*	−1.165	5.14 × 10^−9^	0.42	0.17
*AGFG2*	−1.126	5.69 × 10^−16^	/	/
*PAIP2B*	−1.065	5.1 × 10^−27^	0.29	0.5
*ABCG1*	1.008	8.69 × 10^−11^	/	/
*HOXC11*	1.144	1.79 × 10^−15^	−0.14	−0.11
*SYT7*	1.162	4.75 × 10^−3^	/	/
*CMTM3*	1.381	3.08 × 10^−13^	/	/
*SLC7A8*	1.626	8.78 × 10^−10^	/	/
*EIF2AK2*	1.644	1.34 × 10^−20^	−0.18	−0.19
*WDR72*	1.693	1.08 × 10^−4^	/	/
*CTSC*	1.72	1.88 × 10^−23^	−0.14	−0.16
*NPNT*	1.869	5.04 × 10^−17^	/	/
*PDPN*	2.566	1.09 × 10^−19^	/	/

## Supplementary Materials

The following are available online at https://www.mdpi.com/2079-7737/9/11/379/s1. Figure S1. “Molecular Function” GO enrichment of the 147 miRNA-targeted genes identified through miRCancerdb, Figure S2. “Biological Process” GO enrichment of the 147 miRNA-targeted genes identified through miRCancerdb, Figure S3. “Protein Class” GO enrichment of the 147 miRNA-targeted genes identified through miRCancerdb, Figure S4. GEPIA analysis of the miRNA-targeted genes differentially expressed between oral cancer and healthy donors, Figure S5. Panel (A) No statistical difference observed for hsa-miR-133a-3p expression between oral cancer patients with positive lymph nodes and oral cancer patients without lymph nodes involvement according to the TCGA HNSC data; Panel (B) No statistical difference observed for hsa-miR-375-3p expression between oral cancer patients with positive lymph nodes and oral cancer patients without lymph nodes involvement according to the TCGA HNSC data.
