# Supplementary material for: Droplet Digital PCR Analysis of Liquid Biopsy Samples Unveils the Diagnostic Role of hsa-miR-133a-3p and hsa-miR-375-3p in Oral Cancer
Source: Biology (Basel). 2020 Nov 6;9(11):379. doi: 10.3390/biology9110379 (PMC7694750; doi:10.3390/biology9110379)
Supplement: Supplementary file 1 [file biology-09-00379-s001.pdf]

biology MDPI

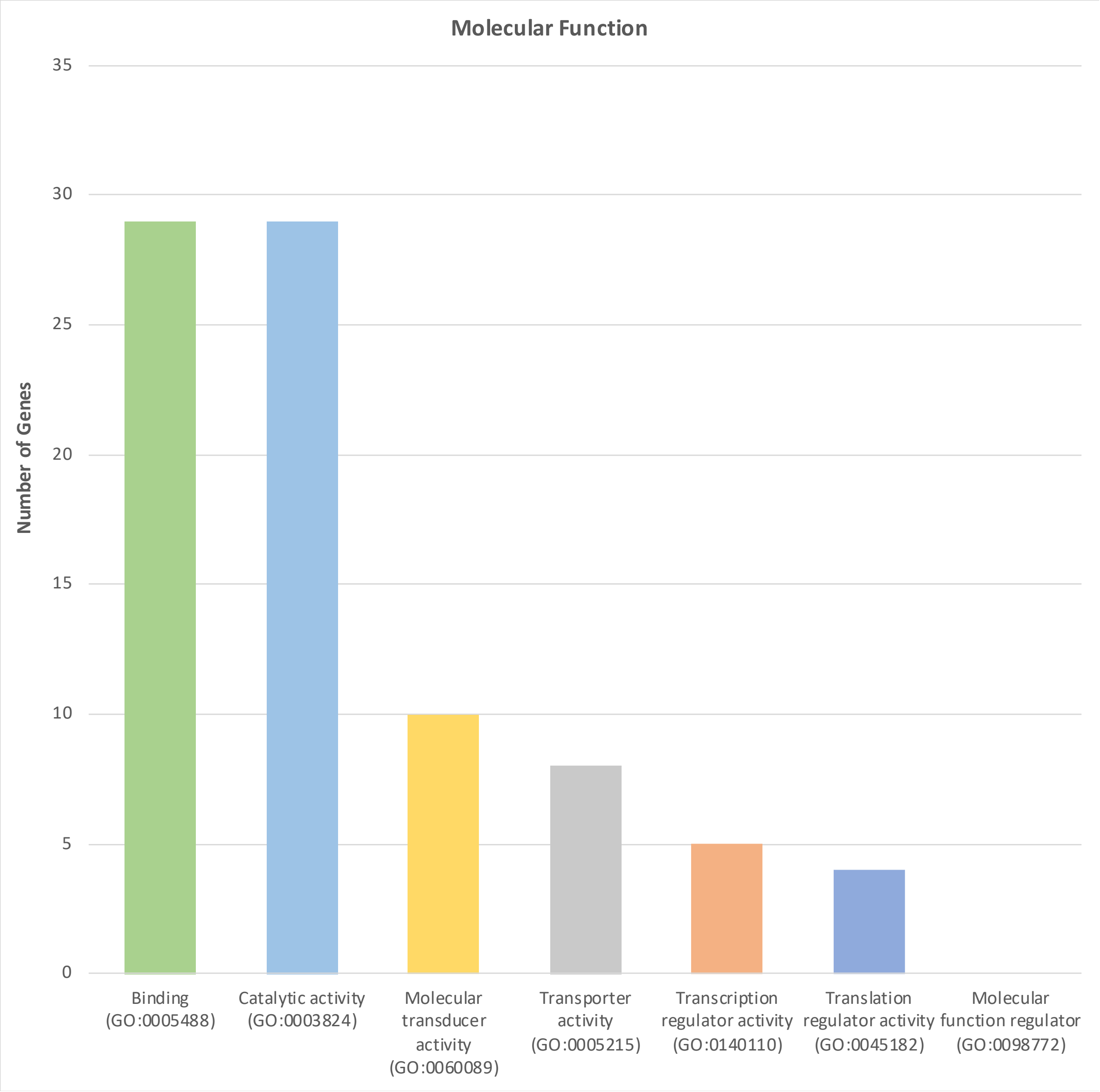


**Figure S1.** "Molecular Function" GO enrichment of the 147 miRNA-targeted genes identified through miRCancerdb.

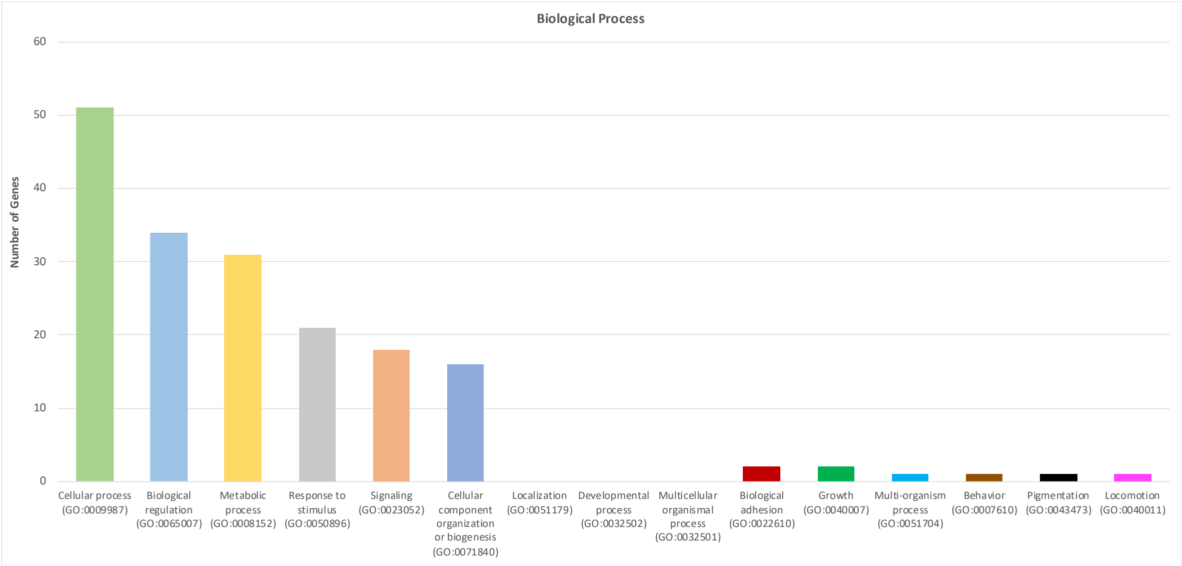


**Figure S2.** "Biological Process" GO enrichment of the 147 miRNA-targeted genes identified through miRCancerdb.

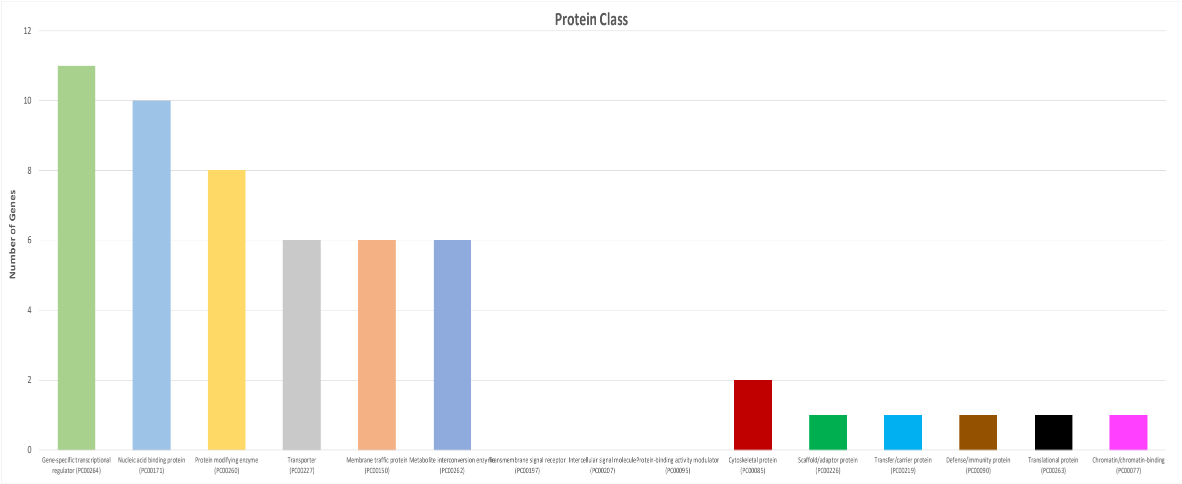


**Figure S3.** "Protein Class" GO enrichment of the 147 miRNA-targeted genes identified through miRCancerdb.

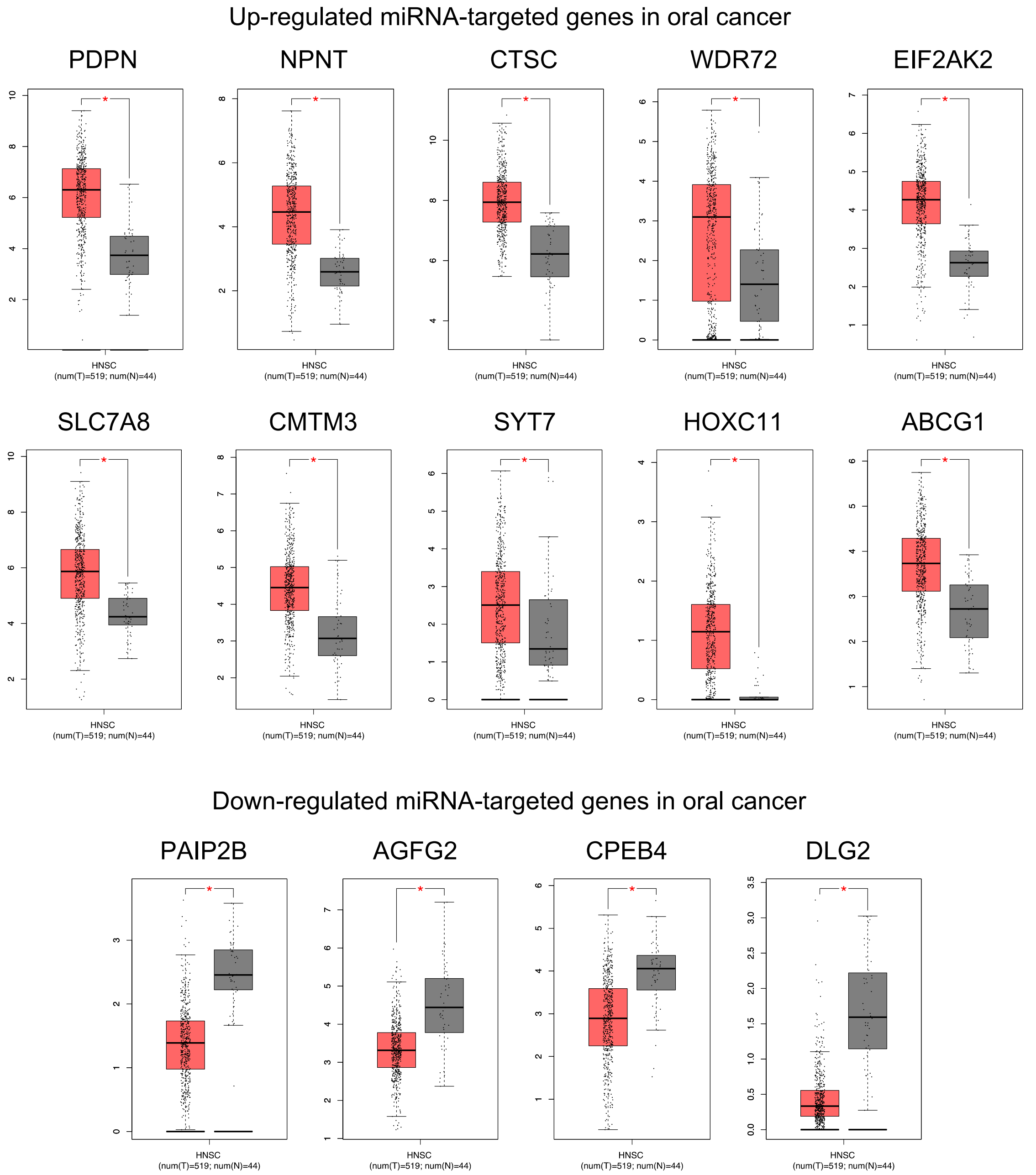


**Figure S4.** GEPIA analysis of the miRNA-targeted genes differentially expressed between oral cancer and healthy donors.

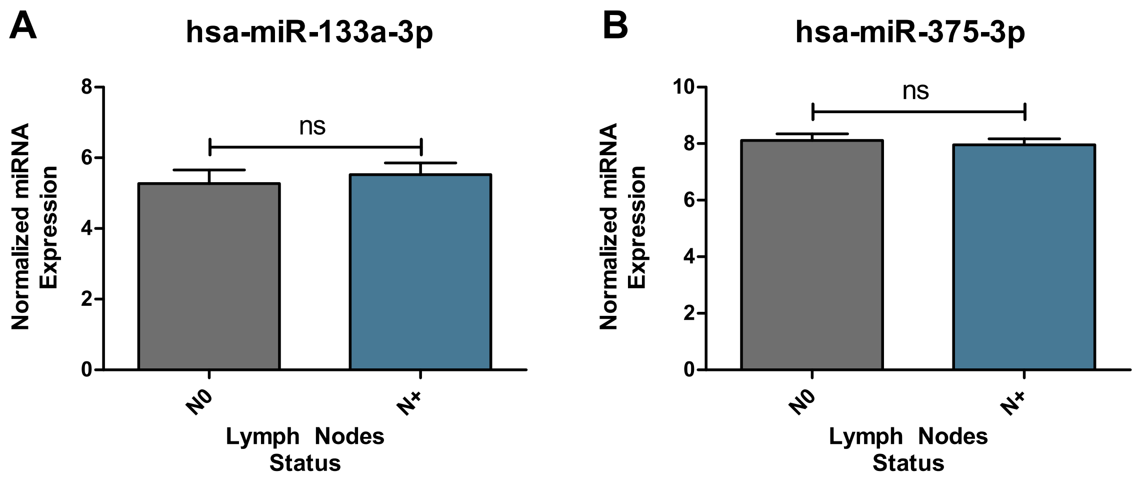


**Figure S5.** Panel A) No statistical difference observed for hsa-miR-133a-3p expression between oral cancer patients with positive lymph nodes and oral cancer patients without lymph nodes involvement according to the TCGA HNSC data; Panel B) No statistical difference observed for hsa-miR-375-3p expression between oral cancer patients with positive lymph nodes and oral cancer patients without lymph nodes involvement according to the TCGA HNSC data.

**Publisher's Note:** MDPI stays neutral with regard to jurisdictional claims in published maps and institutional affiliations.

© 2020 by the authors. Licensee MDPI, Basel, Switzerland. This article is an open access article distributed under the terms and conditions of the Creative Commons Attribution (CC BY) license (http://creativecommons.org/licenses/by/4.0/).
